# Variation in Experiences and Attainment in Surgery Between Ethnicities of UK Medical Students and Doctors (ATTAIN): Protocol for a Cross-Sectional Study

**DOI:** 10.2196/40545

**Published:** 2023-06-16

**Authors:** Samar Babiker, Innocent Ogunmwonyi, Maria W Georgi, Lawrence Tan, Sharmi Haque, William Mullins, Prisca Singh, Nadya Ang, Howell Fu, Krunal Patel, Jevan Khera, Monty Fricker, Simon Fleming, Lolade Giwa-Brown, Peter A Brennan, Ekpemi Irune, Stella Vig, Arjun Nathan

**Affiliations:** 1 St Mary's Hospital London United Kingdom; 2 Darent Valley Hospital London United Kingdom; 3 University College London Medical School London United Kingdom; 4 Tunbridge Wells Hospital Kent United Kingdom; 5 Faculty of Health, Medicine and Life Sciences Maastricht University Maastricht Netherlands; 6 St Georges University Hospital National Health Service Trust London United Kingdom; 7 University of Birmingham Medical School Birmingham United Kingdom; 8 Barts and The London School of Medicine and Dentistry London United Kingdom; 9 South West London Elective Orthopaedic Centre London United Kingdom; 10 University Hospital Coventry & Warwickshire Coventry United Kingdom; 11 Imperial College London London United Kingdom; 12 Institute of Health Sciences Education Queen Mary University of London London United Kingdom; 13 Barts Health National Health Service Trust Royal London Hospital London United Kingdom; 14 Maxillofacial Unit Queen Alexandra Hospital Portsmouth United Kingdom; 15 Department of Otolaryngology, Head and Neck Surgery Cambridge University Hospitals National Health Service Foundation Cambridge United Kingdom; 16 Department of Vascular and General Surgery Croydon University Hospital London United Kingdom; 17 Division of Surgery and Interventional Sciences University College London London United Kingdom

**Keywords:** diversity in surgery, Black and Minority Ethnic, BME in surgery, differential attainment, diversity, surgery, health care system, surgical training, disparity, ethnic disparity, ethnicity, medical student, doctor, training experience, surgical placements, physician, health care provider, experience, perception, cross-sectional, doctor in training, resident, fellow, fellowship, questionnaire, survey, Everyday Discrimination Scale, Maslach Burnout Inventory, Higher Education, ethnicities

## Abstract

**Background:**

The unequal distribution of academic and professional outcomes between different minority groups is a pervasive issue in many fields, including surgery. The implications of differential attainment remain significant, not only for the individuals affected but also for the wider health care system. An inclusive health care system is crucial in meeting the needs of an increasingly diverse patient population, thereby leading to better outcomes. One barrier to diversifying the workforce is the differential attainment in educational outcomes between Black and Minority Ethnic (BME) and White medical students and doctors in the United Kingdom. BME trainees are known to have lower performance rates in medical examinations, including undergraduate and postgraduate exams, Annual Review of Competence Progression, as well as training and consultant job applications. Studies have shown that BME candidates have a higher likelihood of failing both parts of the Membership of the Royal Colleges of Surgeons exams and are 10% less likely to be considered suitable for core surgical training. Several contributing factors have been identified; however, there has been limited evidence investigating surgical training experiences and their relationship to differential attainment. To understand the nature of differential attainment in surgery and to develop effective strategies to address it, it is essential to examine the underlying causes and contributing factors. The Variation in Experiences and Attainment in Surgery Between Ethnicities of UK Medical Students and Doctors (ATTAIN) study aims to describe and compare the factors and outcomes of attainment between different ethnicities of doctors and medical students.

**Objective:**

The primary aim will be to compare the effect of experiences and perceptions of surgical education of students and doctors of different ethnicities.

**Methods:**

This protocol describes a nationwide cross-sectional study of medical students and nonconsultant grade doctors in the United Kingdom. Participants will complete a web-based questionnaire collecting data on experiences and perceptions of surgical placements as well as self-reported academic attainment data. A comprehensive data collection strategy will be used to collect a representative sample of the population. A set of surrogate markers relevant to surgical training will be used to establish a primary outcome to determine variations in attainment. Regression analyses will be used to identify potential causes for the variation in attainment.

**Results:**

Data collected between February 2022 and September 2022 yielded 1603 respondents. Data analysis is yet to be competed. The protocol was approved by the University College London Research Ethics Committee on September 16, 2021 (ethics approval reference 19071/004). The findings will be disseminated through peer-reviewed publications and conference presentations.

**Conclusions:**

Drawing upon the conclusions of this study, we aim to make recommendations on educational policy reforms. Additionally, the creation of a large, comprehensive data set can be used for further research.

**International Registered Report Identifier (IRRID):**

DERR1-10.2196/40545

## Introduction

### Overview

Diversity and inclusion in the health care workforce is fundamentally important to the delivery of health care. There is strong evidence to suggest that an inclusive workforce is able to better meet the needs of a diverse patient population and leads to better patient outcomes [1,2]. According to the latest available data from National Health Service Digital, as of September 2021, the share of Black and Minority Ethnic (BME) staff in the National Health Service workforce in England was 24.2%, which is higher than the proportion of BME people in the general population (18%) [3].

One of the current barriers to a diverse workforce is differential attainment. This is defined as “an unexplained variation in results in assessment, training, and recruitment outcomes seen in candidates based on factors other than academic ability when compared to their peers” [4] and is presently observed across several important demographic characteristics, including gender, age, socioeconomic status, and ethnicity. It is well recognized that Asian and BME trainees are more likely to perform poorly in undergraduate and postgraduate medical examinations [5,6], Annual Review of Competence Progression, and applications for training and consultant posts [7,8]. A BME candidate is independently more likely to fail both part A and Part B of the Membership of the Royal Colleges of Surgeons (MRCS) examinations and 10% less likely to be deemed appointable for core surgical training [9,10]. Additionally, only 20% of leadership roles across the surgical colleges are held by BME surgeons [11] despite BME staff making up 46% of the medical workforce [12]. Furthermore, BME surgeons are disproportionately more likely to be referred to the General Medical Council (22% BME vs 17% White) [10].

The current understanding of causes of differential attainment is derived from thematic analyses on the barriers and issues facing BME doctors. There are several recognized factors, including curriculum and examination biases; relationships between BME trainees and trainers; a lack of support and mentorship; differences in social and cultural capital; and feelings of workplace exclusion due to microaggressions, harassment, and discrimination [13-17]. The Royal College of Surgeons of England’s Kennedy Report specifically attributes these factors as potential barriers to the progression of equality and diversity in surgery [10]. This is significant as surgical training has a unique reliance on the “apprenticeship” between the trainee and the trainer, leading to potentially unique differences in the relative effect of the aforementioned factors associated with differential attainment in surgery.

### Rationale for This Study

The evidence base for determining differential attainment is generated by smaller scale, qualitative studies, which have demonstrated that the experience of BME students and trainees is a factor contributing to differential attainment. However, smaller-scale studies may only capture a limited insight of the experiences of BME trainees and provide limited appreciation of the heterogeneous mix of different trainees of different ethnicities with a variety of experiences under the BME umbrella. Additionally, there is little published evidence that focuses on assessing the unique elements of the “surgical” experiences of surgical trainees, junior doctors, and medical students. This observational, multicenter study aims to compare both the experiences of surgical training between different ethnicities and the level of attainment reached. This study will ultimately aim to evaluate the relationship between educational and training experiences in surgery and attainment and analyze the role of ethnicity in this relationship.

### Aims and Objectives

The ATTAIN (Variation in Experiences and Attainment in Surgery Between Ethnicities of UK Medical Students and Doctors) study aims to answer the following questions:

- Are the experiences and perceptions of surgical education different between BME and non-BME medical students and junior doctors? If so, how and where are these differences seen?
- In what ways are measures of attainment in surgery linked to experiences and perceptions of surgical education?

#### Primary Objective

The primary objective of this study is as follows:

- To compare the effect of experiences and perceptions of surgical education of students and doctors of different ethnicities.

#### Secondary Objectives

The secondary objectives of this study are the following:

- To measure potential differences in both the experiences and perceptions of surgical education of students and doctors of different ethnicities on attainment.
- To identify potential factors associated with potential differences in educational experience and attainment between the aforementioned cohorts as areas of further study.

## Methods

This study will consist of a 15-minute questionnaire (Multimedia Appendix 1), which will be administered over a 28- to 32-week period. This will be distributed to every medical school and Higher Education England region.

Our population of interest consists of all medical students and nonconsultant grade doctors, including doctors in training and nontraining roles in foundation year, core or specialty trainee, as well as fellowship or staff and associate specialist (SAS) grade equivalents, with any experience of surgical training in the United Kingdom. This will be defined as any substantial period of time (being more than a total of 4 weeks) when the primary place of work or learning was (or is) in a surgical department or specialty, including but not limited to medical school rotations, assistantships, and clinical roles.

### Inclusion Criteria

The following will be included in the study:

- Medical students at UK medical schools, identified by the Medical Schools Council, who have self-identified as having experience of surgical training as defined above.
- Anyone who has worked as a nonconsultant grade doctor (including doctors in training and nontraining roles in foundation year, core or specialty trainee, as well as fellowship or staff and SAS grade equivalents) in the United Kingdom, regardless of the country of graduation with experience of surgical training as defined above, either during medical school (if graduated in the United Kingdom) or when working as a doctor in the United Kingdom.

### Exclusion Criteria

The following are excluded from the study:

- Doctors or medical students who have not had either a surgical placement during medical school or experience of surgical training in the United Kingdom as defined above.
- Doctors or medical students who have not either attended medical school in the United Kingdom or worked in the United Kingdom in a clinical role as a doctor.
- Consultant doctors who have completed their surgical training, attained their certificate of completion of training, and are employed as a temporary or permanent consultant.

### Survey Development

The survey was developed using the guidance set out in “Developing questionnaires for educational research: AMEE Guide No. 87” [18]. An initial literature review was conducted based on the General Medical Council’s “Evaluating the impact of interventions aimed at addressing variation in progression associated with protected characteristics known as Differential Attainment” [19]. Expert opinion was sought from academic surgeons and medical educationalists (Katherine Woolf, PAB, SF, EI, and SV) to increase content validity. Questions were integrated from previously validated questionnaires, such as the Everyday Discrimination Scale [20] and Maslach Burnout Inventory [21], to increase the construct validity of our survey. A further focus group was conducted to ensure the conceptualization of our constructs matched how respondents thought of them, to increase the face validity of our questionnaire. A pilot questionnaire (N=78) was conducted to ensure reliability and to determine construct validity. This is a first of its kind cross-sectional survey; therefore, we will be using the preliminary survey with results as a comparator to assess reliability of the study. In addition, questions were based on literature and evidence-based theories that underwent peer review from established experts in this field. Our questionnaire will collect demographic data, including questions on protected characteristics such as self-defined ethnicity, socioeconomic status, gender, and grade. Data on academic attainment will consist of questions detailing a participant’s achievements, including medical school decile, prizes, publications, Annual Review of Competence Progression outcomes, MRCS examination results, and specialty training application outcomes. Surgical experiences will consist of questions around the working relationships participants have with colleagues, experiences of discrimination due to ethnicity, and feelings of burnout.

### Patient and Public Involvement Statement

This study will involve input of medical students and doctors of different grades. An initial survey was distributed at a national medical conference, which yielded information on topics of interest. The preliminary questionnaire was piloted among medical students and junior doctors, who gave qualitative feedback on the design of the study. The finalized questionnaire was distributed to collaborators who gave additional feedback.

### Data Collection

Eligible participants will be invited to participate via web-based and in-person recruitment, including but not limited to medical school societies, medical school mailing lists, organizations focused on medical education, social media, surgical colleges, Higher Education England deaneries, and independent professional surgical bodies.

Additionally, snowball sampling will be incorporated, as eligible participants will be invited to collaborate as regional leads prior to the study period, with one collaborator per medical school or deanery. The collaborators will ensure that their medical school or training deanery is engaged with the study; they will be responsible for dissemination of the questionnaire and are recruited specifically to ensure participants are representative of their local population to prevent bias. Eligible regional leads will be acknowledged as collaborative authors under the “ATTAIN Study Group” in future publications. Participants will also be asked to share the questionnaire with other eligible colleagues.

### Data Protection, Consent, and Confidentiality

The questionnaire will be hosted and stored in Jisc [22], which is compliant with regulations including General Data Protection Regulation and good clinical practice guidelines (ICH E6). The research team will have access to data determined by the study lead. Audit logs are also available to provide an overview of data access and modifications. Participants will receive a participant information sheet before starting the questionnaire. Participants will give consent for publication on completion of the questionnaire; consent can be rescinded up until completion of the questionnaire for any or no reason. On completion and submission of the questionnaire, consent cannot be withdrawn, as no identifiable data are collected. If the questionnaire is not completed, data will not be collected or stored. The only personal data collected from participants is ethnicity, participants’ names or email addresses will not be collected. Any data collected will only be accessed by the members of the research team at any time. Upon the completion of each questionnaire, all documentation will be stored on a secure server that will be only accessible to the chief investigator, study leads, and statistics team.

### Data Analysis

#### Statistical Analysis

The Strengthening the Reporting of Observational Studies in Epidemiology (STROBE) [23] guidelines for cross sectional studies will be followed. Participants will be stratified by level of training prior to analysis, and results will be presented separately. Methods of data imputation will not be used. Descriptive statistics will be presented by ethnicity. Demographic characteristics and questionnaire responses will be described by the following statistics: mean (SD), median (IQR), and frequency with proportion. All hypotheses will be tested at a 5% significance level. Differences in participant characteristics and questionnaire responses between ethnicities will be assessed using parametric and nonparametric tests, including chi-square tests, ANOVA, and Kruskal-Wallis H tests, which will be followed by multiple appropriate post hoc pairwise comparisons with Bonferroni correction. An explanatory model will be created by hierarchical multiple linear regression, with the dependent variable attainment and the following covariates: ethnicity, perceptions of surgical training, surgical experience, sex, socioeconomic status, and age. A correlation matrix will test for the presence of multicollinearity. Another possible confounding variable will be geographical location; the data will be stratified by region and examined for such an effect for those regions with a sufficient number of respondents. SPSS (version 28; IBM Corp) [24] will be used for analysis. Post hoc analyses may be undertaken in the future as part of follow-up studies.

#### Sample Size Calculation

A priori sample size calculation was performed for a 2-tailed test of 2 independent proportions using G*Power (version 3.1.9.7; Heinrich-Heine-Universität Düsseldorf) [25], based on a comparison of MRCS A pass rates between White and BME candidates. Our predicted effect size was estimated based on historical data sourced by Scrimgeour et al [9] from the Intercollegiate MRCS database held by the Royal College of Surgeons of England. With α and β error probability set to .05 and .2 respectively, a minimum of 318 participants per group will be required. Broadly stratifying by level of training (eg, medical student, foundation or core trainee, and registrar or SAS grade) and dividing into BME versus non-BME, this means that 1908 participants will be required (318 × 6 = 1908).

### Ethics Approval

The protocol was approved by the University College London Research Ethics Committee on September 16, 2021 (19071/004). Participants gave informed consent for their anonymized responses to be used for research purposes, including for analysis, presentation, and publication of data. There will be no compensation type for participation in the study.

## Results

Data collected between February 2022 and September 2022 yielded 1603 respondents. Data analysis is yet to be competed. The findings will be disseminated through peer-reviewed publications and conference presentations.

## Discussion

### Overview

#### Impact

Understanding the combined effect of individual experiences of surgical training is important in breaking barriers and promoting change within the medical workforce, leading to a more diverse and inclusive profession. To our knowledge, this is the first study to evaluate the relationship between educational and training experiences in surgery and attainment and analyze the role of ethnicity in this relationship. Although both the presence of [7-17] and possible causative factors for differential attainment based on gender, ethnicity, and socioeconomic status have been demonstrated [26-28], a comprehensive, high-powered data set linking experience and attainment is not currently present.

#### Expected Impact

The ATTAIN study’s findings will add value to the current body of work in differential attainment by providing further insight and context to the interaction between different causative factors. We also hope to highlight areas of excellence and areas of improvement in surgical education, directing suggestions for evidence-based changes to undergraduate and postgraduate surgical education. Our findings offer policy makers evidence-informed recommendations to improve undergraduate and postgraduate surgical education, which will guide the creation and expansion of resources that can efficiently mitigate differential attainment. As society moves to tackle systemic inequalities, we hope our study will demonstrate a commitment to achieve this within medicine and surgery.

We also hope to remind the workforce of the principles of respect that are pivotal to our duties as health care professionals. Mental well-being and burnout are key performance indicators and should be protected to drive excellence in an individual's career progression.

### Limitations

One of the potential limitations of this study is the lack of prior validated questionnaires on differential attainment. It could be argued that the topic of “differential attainment” is so large that a single questionnaire would not be an appropriate method able to collect valid or reliable data. We have tried to improve the quality and rigor of our questionnaire by using a well-established methodology for questionnaire construction and incorporating elements from previously validated questionnaires that are valid for our research question. However, bias cannot be eliminated from the study. Sampling bias may occur if recruitment is limited to streams disproportionately used by one group (eg, overusing social media may attract more medical students and junior trainees). This will be minimized by using multiple communication channels, including professional bodies and trainee-led organizations. Additionally, recall bias is unavoidable in sections of the questionnaire where past achievements are recounted.

We also recognize that our research question is heavily focused on ethnicity; however, there are several different factors that are known to be independently associated with differential attainment, including gender and socioeconomic status.

Although these factors may be controlled for in the study, it may be difficult to completely account for nonlinear intersectional relationships between factors. Future research should be considered to explore the relationships between these different factors in more detail.

### Conclusions

We envisage that the results of the study will provide us with an advancement in knowledge on factors that correlate to attainment and provide us with greater depth in understanding the link between attainment in surgery and experiences of surgical training. Following the results, we aim to create national guidance for independent surgical bodies to incorporate into the current strategies used to tackle the inequalities and injustices in health care.

## Appendix

Multimedia Appendix 1 The ATTAIN (Variation in Experiences and Attainment in Surgery Between Ethnicities of UK Medical Students and Doctors” study survey.

## Abbreviations

BME: Black and Minority Ethnic
MRCS: Membership of the Royal Colleges of Surgeons
SAS: staff and associate specialist
STROBE: Strengthening the Reporting of Observational Studies in Epidemiology

## Data Availability

The data sets generated during and analyzed during this study are available from the corresponding author on reasonable request.
