# Supplementary material for: Variation in Experiences and Attainment in Surgery Between Ethnicities of UK Medical Students and Doctors (ATTAIN): Protocol for a Cross-Sectional Study
Source: JMIR Res Protoc. 2023 Jun 16;12:e40545. doi: 10.2196/40545 (PMC10337414; doi:10.2196/40545)
Supplement: Multimedia Appendix 1 [file resprot_v12i1e40545_app1.pdf]

# ATTAIN - A cross sectional study comparing experiences and attainment in surgery between ethnicities of UK medical students and doctors (Questionnaire)

## Page 1: Introduction

**Attain is cross sectional study comparing experiences and attainment in surgery between ethnicities of UK medical students and doctors (ATTAIN).**

# Our aim is to better understand differential attainment in surgery and improve opportunities for all.

**Background:** Literature shows that Black, Asian and minority ethnic doctors (BAME) are more likely to fail examinations, have unfavourable ARCP outcomes and are required to apply for more consultancy jobs. Racial inequality is rife in the surgical profession. BAME doctors make up 41.9% of the NHS however BAME surgeons occupy less than 20% of leadership roles with less than 1% being held by black surgeons. We will evaluate the experience, perceptions and attainment within a surgical career for ethnic minorities, as well as comparing between ethnic groups.

**Inclusion Criteria**

- Medical students at UK medical schools identified by the Medical Schools Council who have self-identified as having experience of surgical training as defined above.
- Anyone who has worked as a non-consultant grade doctor (including doctors in training and non-training roles in foundation year, core/specialty trainee, fellowship or staff and associate specialist (SAS) grade equivalents in the U.K), in the U.K, regardless of the country of graduation with experience of surgical training as defined either
    - During medical school (if graduated in the U.K)
    - Working as a doctor in the U.K.

**Exclusion Criteria**

- Doctors or medical students who have NOT had either a surgical placement during medical school OR experience of surgical training in the U.K as defined above.
- Doctors or medical students who have NOT either attended medical school in the U.K OR worked in the U.K in a clinical role as a doctor.
- Consultant doctors who have completed their surgical training, attained their certificate of completion of training (CCT) and are employed as a temporary or permanent consultant.

**Ethics statement:** This study received ethical approval from the University College London (UCL) ethics committee on 16/09/2021 (Ethics approval reference 19071/004).

**Consent:** Consent is included within the study questionnaire. On completion and submission of the questionnaire consent cannot be withdrawn.

**Data:** If the questionnaire is not completed, data will not be stored. This questionnaire will collect demographic data including self-defined ethnicity, socioeconomic status and gender. Data collected will only be accessed by the members of the research team. Upon the completion of each survey, all documentation will be stored securely on UCL OneDrive in a folder that only the chief investigator, study leads, and statistics team will have access to. The written results will be submitted for publication in a peer-reviewed journal and presented at national/international meetings. A centralised website (https://nsts-ed.com/research-projects/), open to the public, allows access to a list of publications/conferences the ATTAIN study team have been involved with. Data will be archived for a maximum of 3 years after the date of survey closure for further subgroup analysis.

**Study Leads:** Dr Innocent Ogunmwonyi and Dr Samar Babiker

**Chief Investigator:** Dr Arjun Nathan

**Steering Committee:** Professor Katherine Woolf, Professor Peter Brennan, Miss Irune Ekpemi, Mr Simon Fleming, Miss Stella Vig, Miss Lola Giwa

## age 2: Demographic Data

1. Please select your gender *Required*

- ◯ Male
- ◯ Female
- ◯ Non-binary
- ◯ Other

2. Please select your ethnic group *Required*

3. What is the highest level of qualifications achieved by either of your parent(s) or guardian(s) by the time you were 18? *Required*

*a.* Are either of your parent(s) or guardian(s) medical doctors?

- ◯ Yes
- ◯ No

*b.* Before the age of 18, which best describes the sort of work the main/highest income earner in your household did in their main job? *Required*

*c.* What type of school did you mainly attend between the ages of 11 and 16? *Required*

*d.* Please select your average annual household/family income:

- <£12,000
- £12,000 - £20,000
- £20,000 - £30,000
- £30,000 - £50,000
- £50,000 - £80,000
- £80,000 - £100,000
- Above £100,000
- Prefer not to say

*e.* Were you eligible for Free School Meals at any point during your school years? *Required*

*f.* Prior to the age of 18, did you spend time living in care? *Required*

*g.* Have you ever had refugee or asylum status? *Required*

4. Please select your training grade *Required*

## Page 3: Academic Achievements

We are going to ask you a few questions about academic achievement to date. Please try to be as accurate as you can. If you cannot remember, please answer ‘I do not know’.

This part of the survey uses a table of questions, view as separate questions instead?

**5.** If you are currently a medical student, please select the decile you achieved in your last completed year of medical school *Required*

Please don't select more than 1 answer(s) per row.

Please select at least 1 answer(s).

| | 1st (Highest) | 2nd | 3rd | 4th | 5th | 6th | 7th | 8th | 9th | 10th(Lowest) | N/A |
|---|---|---|---|---|---|---|---|---|---|---|---|
| Medical School decile | ☐ | ☐ | ☐ | ☐ | ☐ | ☐ | ☐ | ☐ | ☐ | ☐ | ☐ |

This part of the survey uses a table of questions, view as separate questions instead?

*a.* If you have graduated from medical school, please select your final year decile of medical school *Required*

Please don't select more than 1 answer(s) per row.

Please select at least 1 answer(s).

| | 1st (Highest) | 2nd | 3rd | 4th | 5th | 6th | 7th | 8th | 9th | 10th(Lowest) | N/A |
|---|---|---|---|---|---|---|---|---|---|---|---|
| Graduation Decile | ☐ | ☐ | ☐ | ☐ | ☐ | ☐ | ☐ | ☐ | ☐ | ☐ | ☐ |

**6.** Did you ever have to sit any examinations in medical school more than once? *Required*

- Yes
- No

**7.** Did you ever have to take any placements in medical school more than once? *Required*

- Yes
- No

**8.** If you are a doctor, have you ever had an unfavourable ARCP outcome? (If you are a medical student, please select N/A) *Required*

- Yes
- No
- N/A

**9.** Have you passed MRCS **Part A?** (If you are a medical student, please select N/A) *Required*

- Yes
- No
- N/A

**10.** Have you passed **MRCS Part B?** (If you are a medical student, please select N/A) *Required*

- Yes
- No
- N/A

*b.* Have you applied for specialty training in surgery? *Required*

YES, but I have NOT been successful

YES, and I have been successful

NO

This part of the survey uses a table of questions, view as separate questions instead?

11. From the start of medical school to date, please select the number of your achievements below. (Numerical = 0, 1, 2, 3, 4, 5+) *Required*

Please don't select more than 1 answer(s) per row.

Please select at least 6 answer(s).

| | 0 | 1 | 2 | 3 | 4 | 5 | 6+ |
|---|---|---|---|---|---|---|---|
| Merits/distinctions | ☐ | ☐ | ☐ | ☐ | ☐ | ☐ | ☐ |
| Prizes | ☐ | ☐ | ☐ | ☐ | ☐ | ☐ | ☐ |
| Poster and oral presentations as an author(national or international) | ☐ | ☐ | ☐ | ☐ | ☐ | ☐ | ☐ |
| Poster and oral presentations as a presenter(national or international) | ☐ | ☐ | ☐ | ☐ | ☐ | ☐ | ☐ |

| | | | | | | | |
|---|---|---|---|---|---|---|---|
| Papers authored as a first author with PubMed indexed original research | ☐ | ☐ | ☐ | ☐ | ☐ | ☐ | ☐ |
| Papers authored as a co-author with PubMed indexed original research | ☐ | ☐ | ☐ | ☐ | ☐ | ☐ | ☐ |
| One cycle of a surgically themed audits/quality improvement project | ☐ | ☐ | ☐ | ☐ | ☐ | ☐ | ☐ |
| Two cycles of a surgically themed audits/quality improvement projects | ☐ | ☐ | ☐ | ☐ | ☐ | ☐ | ☐ |

## Page 4: Surgical Experiences

The following questions are about your experiences whilst in surgery. This includes any placements, electives or SSCs undertaken as either an undergraduate or postgraduate to date.

**12.** During medical school, select what you have participated to date: *Required*

- ☐ A surgically themed elective
- ☐ A surgically themed student selected component
- ☐ A surgically themed intercalation/additional degree
- ☐ None

**13.** As a graduate, select what you have participated in to date: *Required*

- ☐ A surgically themed taster week
- ☐ A surgically themed additional degree
- ☐ None

This part of the survey uses a table of questions, view as separate questions instead?

**14.** To what extent do you agree with the following statements? 1 = Strongly Disagree, 2 = Disagree, 3 = Neutral, 4 = Agree, 5 = Strongly Agree *Required*

Please don't select more than 1 answer(s) per row.

Please select at least 8 answer(s).

| | 1 = Strongly Disagree | 2 = Disagree | 3 = Neutral | 4 = Agree | 5 = Strongly Agree |
|---|---|---|---|---|---|
| My relationships with senior colleagues/placement supervisors in surgery (e.g. consultants, registrars) has been positive overall | ☐ | ☐ | ☐ | ☐ | ☐ |
| My senior colleagues/placement supervisors have provided opportunities for me to attend theatre | ☐ | ☐ | ☐ | ☐ | ☐ |
| When in theatre, my senior colleagues/placement supervisors provide me with the opportunity to get involved/assist | ☐ | ☐ | ☐ | ☐ | ☐ |
| When I get involved in cases, senior colleagues/placement supervisors provide constructive feedback in a respectful manner | ☐ | ☐ | ☐ | ☐ | ☐ |

| | | | | | |
|---|---|---|---|---|---|
| I feel comfortable going to theatre | ☐ | ☐ | ☐ | ☐ | ☐ |
| My senior colleagues/placement supervisors provide opportunities for me to become involved in QI/Audit | ☐ | ☐ | ☐ | ☐ | ☐ |
| My senior colleagues/placement supervisors provide opportunities for me to become involved in research | ☐ | ☐ | ☐ | ☐ | ☐ |
| My senior colleagues/placement supervisors have encouraged me to take up extra responsibilities in my training (e.g leadership roles) | ☐ | ☐ | ☐ | ☐ | ☐ |

15.Within surgery, do you currently have someone who you would consider to be a mentor? *Required*

- ○ Yes
- ○ No

This part of the survey uses a table of questions, view as separate questions instead?

16. How important do you believe having a mentor is in surgical training? *Required*

Please don't select more than 1 answer(s) per row.

Please select at least 1 answer(s).

| | 1 = Not important at all | 2 = Quite unimportant | 3 = Neither important or unimportant | 4 = Quite Important | 5 = Very Important |
|---|---|---|---|---|---|
| Importance of surgical mentors | ☐ | ☐ | ☐ | ☐ | ☐ |

This part of the survey uses a table of questions, view as separate questions instead?

17. Please rate the following statements (1 = Not important at all, 5 = Very important) How important is it that a mentor: *Required*

Please don't select more than 1 answer(s) per row.

Please select at least 5 answer(s).

| | 1 = Not important at all | 2 = Quite unimportant | 3 = Neither important or unimportant | 4 = Quite Important | 5 = Very Important |
|---|---|---|---|---|---|
| Is in a similar speciality to you | ☐ | ☐ | ☐ | ☐ | ☐ |

| | | | | | |
|---|---|---|---|---|---|
| Has a similar ethnic background to you | ☐ | ☐ | ☐ | ☐ | ☐ |
| Is the same gender as you | ☐ | ☐ | ☐ | ☐ | ☐ |
| Is in the same field that you would like to go into | ☐ | ☐ | ☐ | ☐ | ☐ |
| Provides pastoral mentorship | ☐ | ☐ | ☐ | ☐ | ☐ |

## Page 5: Microaggressions at work/university

This part of the survey uses a table of questions, view as separate questions instead?

18. During your surgical placements/rotations, how often have any of the following things happened to you? (1 = Never, 2 = Rarely, 3 = Occasionally, 4 = Frequently, 5 = Very Frequently) *Required*

Please don't select more than 1 answer(s) per row.

Please select between 1 and 5 answers.

| | 1 = Never | 2 = Rarely | 3 = Occasionally | 4 = Frequently | 5 = Very Frequently |
|---|---|---|---|---|---|
| You have felt uncomfortable, undermined, or insulted by someone responding to your race or ethnicity | ☐ | ☐ | ☐ | ☐ | ☐ |
| A co-worker (senior or colleague at an equivalent or junior grade) has used racial or ethnic slurs or jokes at you or others | ☐ | ☐ | ☐ | ☐ | ☐ |

| | | | | | |
|---|---|---|---|---|---|
| You feel like you have to work harder than other colleagues to receive the same level of recognition on account of your ethnicity | ☐ | ☐ | ☐ | ☐ | ☐ |
| You feel that you are ignored or not taken seriously by senior colleagues on account of your ethnicity | ☐ | ☐ | ☐ | ☐ | ☐ |
| You feel as though you have been unfairly humiliated in front of others at work on account of your ethnicity | ☐ | ☐ | ☐ | ☐ | ☐ |

19. If you have witnessed discrimination at work, did you or a colleague report it? *Required*

- ◯ Yes
- ◯ No
- ◯ Prefer not to say

N/A

20. If you did witness an instance of racial or ethic slurs at work, would you be comfortable reporting this/raising concerns? *Required*

Yes

No

Prefer not to say

N/A

21. How do you feel about issues of discrimination within surgical placements?

## Page 6: Feelings of Burnout- Maslach Burnout Inventory
### The next few questions are a measurement for the degree of burnout you have experienced over the past year:

This part of the survey uses a table of questions, view as separate questions instead?

22. To what extent do you agree or disagree with the following statements? (1 = Not at all, 2 = Rarely, 3 = Sometimes, 4 = Often, 5 = Very Often) *Required*

Please don't select more than 1 answer(s) per row.

Please select at least 7 answer(s).

| | 1 = Not at all | 2 = Rarely | 3 = Sometimes | 4 = Often | 5 = Very Often |
|---|---|---|---|---|---|
| I feel/have felt worn out at the end of a day at work or university | ☐ | ☐ | ☐ | ☐ | ☐ |
| I feel/have felt emotionally drained from my surgical placements/rotations | ☐ | ☐ | ☐ | ☐ | ☐ |
| I feel/have felt frustrated by my surgical placements/rotations | ☐ | ☐ | ☐ | ☐ | ☐ |
| I feel/have felt burnt out from my surgical placements/rotations | ☐ | ☐ | ☐ | ☐ | ☐ |

| | | | | | |
|---|---|---|---|---|---|
| I am satisfied with my surgical placements/rotations | ☐ | ☐ | ☐ | ☐ | ☐ |
| I would rate my overall experience in my surgical placements/rotations as positive | ☐ | ☐ | ☐ | ☐ | ☐ |
| I think that during my surgical placements/rotations, respect has been given to individual differences (e.g cultures, working styles, backgrounds, ideas etc) | ☐ | ☐ | ☐ | ☐ | ☐ |

This part of the survey uses a table of questions, view as separate questions instead?

23. How important were the following factors in determining your likelihood to pursue a career in surgery? *Required*

Please don't select more than 1 answer(s) per row.

Please select at least 8 answer(s).

| | 1 = Not important at all | 2 = Unimportant | 3 = Neither important or unimportant | 4 = Important | 5 = Very Important |
|---|---|---|---|---|---|

| | | | | | |
|---|---|---|---|---|---|
| Representation within surgery | ☐ | ☐ | ☐ | ☐ | ☐ |
| Experiences of discrimination | ☐ | ☐ | ☐ | ☐ | ☐ |
| Work - life balance | ☐ | ☐ | ☐ | ☐ | ☐ |
| Opportunity to influence patient care | ☐ | ☐ | ☐ | ☐ | ☐ |
| Influence from mentors | ☐ | ☐ | ☐ | ☐ | ☐ |
| Financial compensation | ☐ | ☐ | ☐ | ☐ | ☐ |
| Professionally challenging | ☐ | ☐ | ☐ | ☐ | ☐ |
| Varied caseload | ☐ | ☐ | ☐ | ☐ | ☐ |

## Page 7: Likeliness to pursue a career in surgery

This part of the survey uses a table of questions, view as separate questions instead?

24. How likely were you to pursue a career in surgery:

Please don't select more than 1 answer(s) per row.

| | 1 = Extremely Unlikely | 2 = Unlikely | 3 = Neither Likely or unlikely | 4 = Likely | 5 = Extremely Likely | N/A |
|---|---|---|---|---|---|---|
| Before medical school | ☐ | ☐ | ☐ | ☐ | ☐ | ☐ |
| Before starting your first surgical rotation in medical school | ☐ | ☐ | ☐ | ☐ | ☐ | ☐ |
| After finishing your surgical rotations in medical school | ☐ | ☐ | ☐ | ☐ | ☐ | ☐ |
| After finishing your surgical rotation in foundation training | ☐ | ☐ | ☐ | ☐ | ☐ | ☐ |

| | | | | | | |
|---|---|---|---|---|---|---|
| Currently | ☐ | ☐ | ☐ | ☐ | ☐ | ☐ |

25. How have your experiences affected your likelihood to pursue a surgical career?

26. How did you hear about the ATTAIN study? *Required*

c. Please give feedback on the study survey here (Comments on question type, who recruited you (if word of mouth), length of questionnaire, other suggestions) *Required*
